# Regulatory effects of COL1A1 on apoptosis induced by radiation in cervical cancer cells

**DOI:** 10.1186/s12935-017-0443-5

**Published:** 2017-07-28

**Authors:** Shurong Liu, Gewang Liao, Guowen Li

**Affiliations:** 1 Department of Gynecologic Oncology, Hunan Cancer Hospital, Tongzipo Road No. 283, Changsha, 410011 Hunan China; 2 Department of Interventional Radiology, Hunan Cancer Hospital, Tongzipo Road No. 283, Changsha, 410011 Hunan China

**Keywords:** COL1A1, Cervical cancer, Radioresistance, Apoptosis

## Abstract

**Background:**

Cervical cancer is a common cancer of women in developing countries, and radiotherapy still remains its predominant therapeutic treatment. Collagen type I alpha 1 (COL1A1) has been shown to have a radioresistance effect in previous studies. However, such effect of COL1A1 has not yet been revealed in cervical cancer.

**Methods:**

Expression of COL1A1 in cervical cancer tissues and normal tissues was assessed by qRT-PCR and immunohistochemistry. The effect of COL1A1 on radioresistance of human cervical cancer cell lines HeLa and CaSki was assessed using the colony formation assay. Apoptosis alterations were analyzed by flow cytometry. In addition, western blotting was used assessed the alterations of several critical apoptosis and signaling pathway related proteins.

**Results:**

The expression of COL1A1 was significantly increased in cervical cancer tissues compared with normal tissues at the mRNA and protein level. Further, based on COL1A1 knock down and COL1A1 activation cell models, a negative correlation was observed between COL1A1 expression level and radiosensitivity. Moreover, the findings are further supported by apoptosis analysis that COL1A1 activation could inhibit the apoptosis of cervical cancer cells. Subsequently, a significantly decreased expression of p-AKT and Bcl-2, increased expression of Caspase-3 were observed in the LY294002 plus radiation group compared with radiation alone group, while these influences caused by LY294002 or X-ray radiation were reversed after COL1A1 activation.

**Conclusions:**

To our knowledge, this is the only study to profile the mechanisms that COL1A1 plays a crucial role in cervical cells anti-apoptosis induced by radiation. Therefore, our identification of radioresistance-related COL1A1 in cervical cancer could be a starting point to explore the function of collagens, adding a new dimension to our understanding of the cervical cancer, assisting cancer biologists and clinical oncologists in novel therapeutic strategies.

**Electronic supplementary material:**

The online version of this article (doi:10.1186/s12935-017-0443-5) contains supplementary material, which is available to authorized users.

## Introduction

Cervical cancer is one of the most common cancers in women worldwide and the second most diagnosed cancer of women in developing countries [1]. The treatment of cervical cancer is primarily based on the stage of disease, and conventional treatments include surgery, chemotherapy, or radiotherapy (RT) [2]. Previous studies have reported that RT is an excellent therapeutic method for cervical cancer patients, especially to the patients with locally advanced disease. However, advanced or metastasized tumors with low radiosensitivity required an increasing radiation dose which may damage the surrounding healthy tissues and organs [3–5]. Therefore, the identification of novel methods to enhance tumor cell radiosensitivity has become a recent focus of medical radiation research [6].

Collagen type I alpha 1 (COL1A1) is a member of group I collagen which include COL1A1 and COL1A2 [7]. Collagen is the main protein of bones, tendons and teeth, and involved in tumor cell adhesion, gap junction and extracellular matrix (ECM) [8–10]. Previous studies have reported that COL1A1 is upregulated in gastric cancer, and plays important roles in cancer cell invasion and metastasis in this cancer [11, 12]. In addition, Kitahara’s cDNA microarray analysis result has indicated that COL1A1 is involved in radioresistance of cervical cancer [13]. Moreover, a recent study confirmed that COL1A1 expression was altered after radiation [14].

In the present study, we hypothesize that COL1A1 is crucial for radioresistance in cervical cancer cells with RT. Here, based on the study of clinicopathological tissues and cell models, we found that COL1A1 played an important role in inhibiting apoptosis induced by radiation in cervical cancer cells. The findings provide a therapeutic target as well as a diagnostic radioresistance in cervical cancer.

## Materials and methods

### Clinical specimens

This study was permitted by the Ethical Committee of our hospital, and obtained consents from all the participants. A total of 20 patients with clinically diagnosed cervical cancer participated in, and their tissues were collected in our Hunan Cancer Hospital. Pathological samples were contained two parts: cervical cancer tissues and tumor adjacent normal tissues.

### Cell culture

The SiHa, CaSki and Hela cell lines were purchased from the Cell Center of the Xiangya School of Medicine, Central South University (Hunan, China), and maintained in high glucose Dulbecco’s modified Eagle’s Eagle (DMEM) (Gibco Life Technologies, USA) containing 10% fetal bovine serum (Gibco Life Technologies, USA) at 37 °C in a 5% CO_2_ incubator.

COL1A1-shRNA and activation plasmids were purchased from (Santa cruz, USA). Following transfection these two plasmids and their negative control plasmids into CaSki and Hela cells respectively.

### Quantitative real time-polymerase chain reaction (qRT-PCR)

The expression of COL1A1 was confirmed by qRT-PCR. Briefly, Total RNA was extracted using total RNA isolation kit (Qiangen, Germany), and PCR was performed by Real Time Quantitative PCR SYBR Green detection reagent (Takara, Japan). The relative expression of COL1A1 was calculated with 2^−ΔΔCT^ method relative to GAPDH. COL1A1 primers: sense, CCC CTG GTG CTA CTG GTT TCC C; antisense, GAC CTT TGC CGC CTT CTT TGC. GAPDH primers: sense, ATT CCA CCC ATG GCA AAT TC; antisense, GAT GGG ATT TCC ATT GAT GAC A. All the procedures were repeated in triplicate.

### Tissue microarray (TMA) and immunohistochemistry (IHC)

Uterine cervical cancer tissue microarrays were purchased from Auragene (Changsha, China). This microarray contained cervical normal tissues (n = 5) and tumor adjacent normal tissues (n = 5) and cancer tissues of different grades (n = 70). The IHC was performed using ElivisionTM plus PolyerHRP (Mouse/Rabbit) IHC Kit (Fuzhou Maixin Biotech, China) according to the manufacturer’s instructions. Briefly, the TMA was baked at 60 °C for 60 min, deparaffinized, and rehydrated through a series of ethanol with different concentrations. The slides were microwaved in Tris/EDTA pH 9.0 buffer solution (10 mM Tris, 1 mM EDTA) for 10 min for antigen retrieval, and then quenched by immersing in 3% hydrogen peroxide in distilled water for 20 min. After blocking the nonspecific binding with 10% normal goat serum in PBS buffer for 15 min, the slides were incubated with anti-Collagen I (1:100 dilution, Abcam, UK) and stored overnight at 4 °C. The slides were sequentially incubated with a secondary antibody (Maxim-Bio, Fuzhou, China). The slides were subsequently treated with 3′3-diaminobenzidine tetra-hydrochloride, counterstained with haematoxylin, and finally mounted with neutral balata. Standardization of the incubation and development times allowed accurate comparisons in all cases. A negative control was obtained by replacing the primary antibody with a normal rabbit IgG.

Sections of tissue were observed under an Olympus microscope (Olympus Corporation, Japan) and images were taken at 400× magnification with the same light intensity and exposure time. All images were then converted to 8-bit grayscale. The COL1A1 staining intensity of cervical tissues was semi-quantitatively compared by analyzing the integrated optical density value of each image, which was measured by the Image-Pro Plus image analysis software (Media Cybernetics, USA).

### Western blotting

The cells were harvested and lysed in lysis buffer, followed by centrifugation at 13,000*g* at 4 °C for 30 min, the supernatants were then collected. After measurement of total protein concentrations using a BCA protein assay kit, equal amounts of total protein (30 μg/sample) were separated by 12% SDS-PAGE, and transferred onto the polyvinylidene difluoride membrane. After blocking in 5% non-fat milk for 1 h, the membranes were incubated overnight at 4 °C with the specific primary antibodies against the following proteins: β-actin (Abcam, UK), and COL1A1, Caspase-3, BAX, Bcl-2, AKT, p-AKT (Cell Signaling Technology, USA). After washing the membranes with Tris-buffered saline, 0.1% Tween 20 for three times, then the membranes was incubated with HRP-conjugated secondary antibody for 1 h at room temperature. The bound antibodies were visualized using chemiluminescence reagents following exposure to X-ray film. All experiments were performed in triplicate. The relative levels of target protein to control β-actin were analyzed by Quantity One 1-D Image Analysis Software (Bio-Rad).

### Radiation

The CaSki and Hela cells were treated with the dose of 0, 2, 4, 6, and 8 Gy in a 6 MV X-rays at a dose rate of 200 cGY/min with a distance to the source irradiation about 100 cm. The cells were further cultured in complete medium for 12 h. The levels of COL1A1 expression were detected by RT-PCR to choose the minimal effective radiation dose of X-ray.

### Cell colony formation assay

Cell colony formation assays were performed using 30 mm cell culture plates coated with 0.5 ml bottom soft agar mixture (DMEM, 20% FBS, 0.6% soft agar). The cells treated with radiation at a dose of 4 Gy were mixed with top agar (DMEM, 20% FBS, 0.3% soft agar) and seeded into each plate, after the bottom layer had solidified. Two weeks later, the colonies were fixed with methanol and stained with 0.5% crystal violet. The number of colonies (>50 cells) was counted on an inverted microscope. The experiments were repeated in triplicate.

### Apoptosis analysis

The CaSki and Hela cells were plated into 60 mm dishes and radiated with the dose of 4 Gy. Twenty-four hours later, the cells were harvested, and then the cellular apoptosis was detected using the Annexin V-FITC Apoptosis Kit (BD Biosciences, USA). The results were examined using the FACS flow cytometer. All these procedures were performed in triplicate.

### Statistical analysis

Quantitative data were expressed as mean ± SD, and analyzed using SPSS 20.0 (IBM, USA). Significant differences between groups were compared using ANOVAs and Student’s *t* test. *P* < 0.05 was considered to be statistically significant.

## Results

### The expression level of COL1A1 is elevated in cervical cancer tissues

**Fig. 1:**
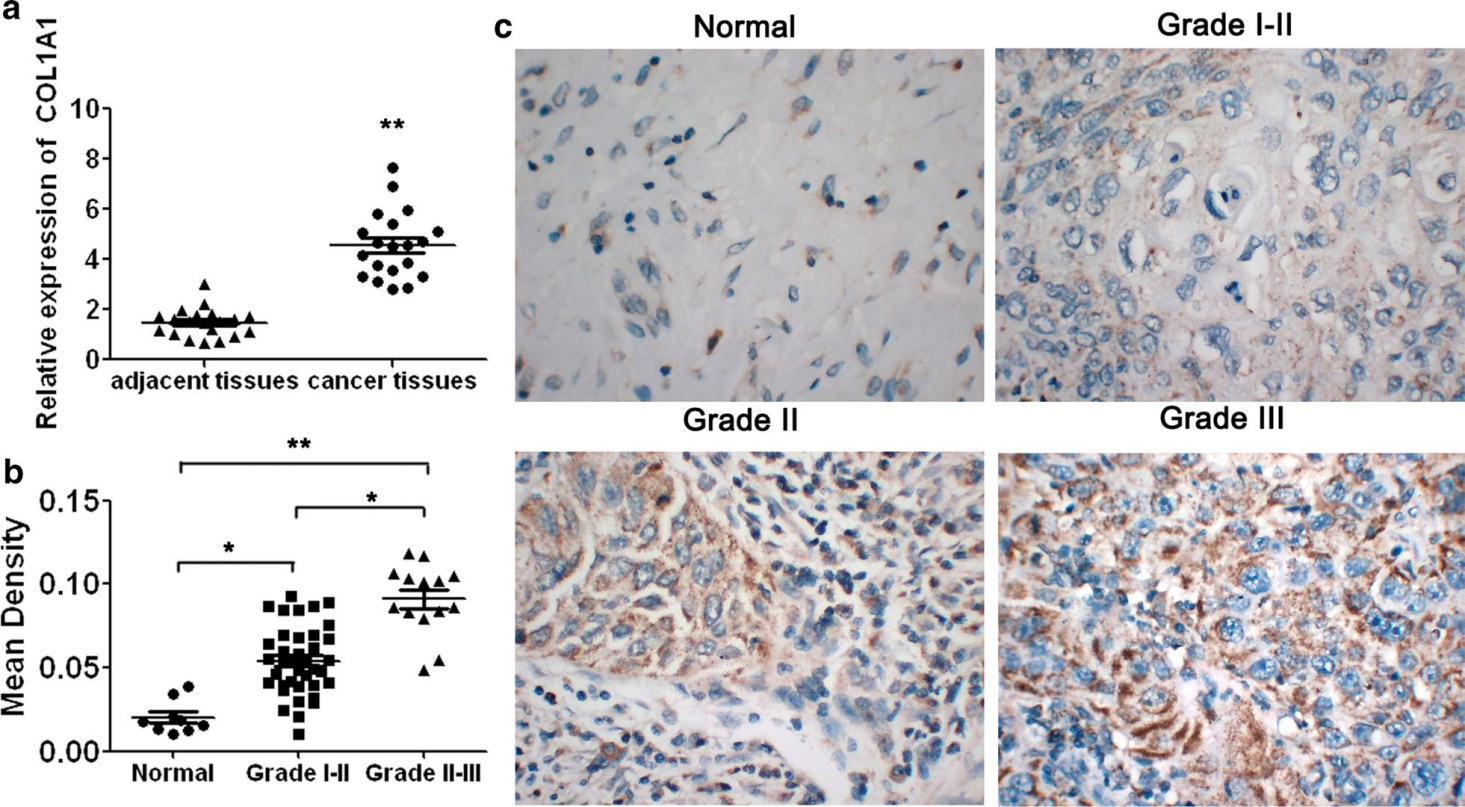
The expression of COL1A1 in cervical normal and cancer tissues. **a** COL1A1 was detected significantly increased in cervical cancer tissues (n = 20) compared with adjacent normal tissues (n = 20). **b** Representative tumor adjacent normal tissue, grade I–II, grade II, grade III cervical cancer tissues sections stained with an antibody against COL1A1, respectively. **c** Optical density measurements indicated that COL1A1 expression from normal cervical specimens to cervical cancer was enhanced gradually. Magnification, ×400. Data are presented as mean ± SEM, *P < 0.05, **P < 0.01

To examine the relationship between COL1A1 expression level and cervical cancer, the difference of COL1A1 mRNA expression levels were measured in 20 patients with cervical cancer between their cervical cancer tissues and adjacent normal tissues by qRT-PCR assay. Our results demonstrated that COL1A1 expression was significant elevated in almost cancer tissues compared to adjacent normal tissues (Fig. 1a, *P* < 0.05). Meanwhile, we determined the COL1A1 protein levels in 70 cervical cancer tissues and 10 normal samples by high throughput IHC analyse. Representative sections of COL1A1 expression in all tissues are shown in Fig. 1b. Obviously, COL1A1 was expressed at substantially higher levels in the cancer samples by contrast to the weak but detectable expression in normal tissues with a statistical significance (*P* < 0.05) (Fig. 1c). Furthermore, a close relationship was observed between the increasing grade of lesion and the intensity of COL1A1 staining in cervical cancer tissues. For COL1A1 IHC staining in cervical cancer, immunoreactivity was primarily observed in the ECM (extracellular matrix) of tumor cells. Therefore, our results suggest that COL1A1 expression was enhanced from normal cervical specimens to different grades cervical cancers gradually.

### The expression of endogenous COL1A1 in cervical cells and constructed of COL1A1 cell models

**Fig. 2:**
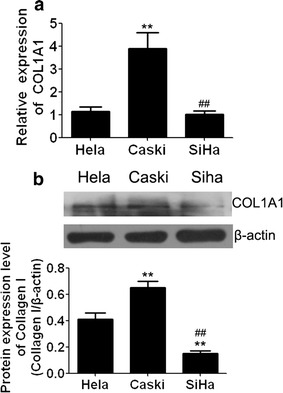
The expression of COL1A1 in Hela, CaSki, SiHa cells and cell models construction. **a** qRT-PCR analysis revealed that COL1A1 is differentially expressed in the cell lines. **b** Differentially expressed of COL1A1 was detected in three different cells by western blotting analysis. **P < 0.01 vs Hela, ^##^ *P* < 0.01 vs Caski

The baseline expression of COL1A1 was explored in cervical cancer cell lines: Hela, CaSki and SiHa (Fig. 2a, b). QRT-PCR and western blotting analysis revealed that COL1A1 was increased expression apparent in Hela and CaSki cells.

**Fig. 3:**
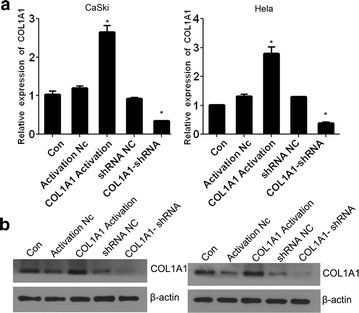
The construction of over expression and interference models. **a** qRT-PCR analysis indicated that COL1A1 expression levels were increased significantly in CaSki and Hela cells with COL1A1 activation plasmids, while COL1A1 expression levels were decreased significantly in CaSki and Hela cells with COL1A1 shRNA plasmids. **b** Protein levels indicated the COL1A1-shRNA and COL1A1 Activation cell models were constructed successfully. *P < 0.05 vs control. Con, cells without plasmid. COL1A1 Activation, cells transfected with COL1A1 Activation plasmids. Activation NC, cells transfected with scramble control of activation plasmid. COL1A1 shRNA, cells transfected with COL1A1 shRNA plasmid. shRNA NC, cells transfected scramble control of shRNA plasmid

Given the progressive up regulation of COL1A1 in human liver and gastric cancers [8], we investigated how COL1A1 influences cellular process by genetically manipulating the expression of COL1A1 in CaSki and Hela cells. COL1A1 knock down and COL1A1 activation cell models were constructed. As shown in Fig. 3a, the expression of COL1A1 in CaSki and Hela cells were increased significantly when transfected with COL1A1 activation plasmids, while, COL1A1 expression levels were decreased significantly when transfected with COL1A1 shRNA plasmids. Moreover, these finding are further supported by western blotting analysis at protein level (Fig. 3b). Thus, the results indicated that stably transfected lines were constructed successfully.

### The effect of COL1A1 in cervical cells with radiation

**Fig. 4:**
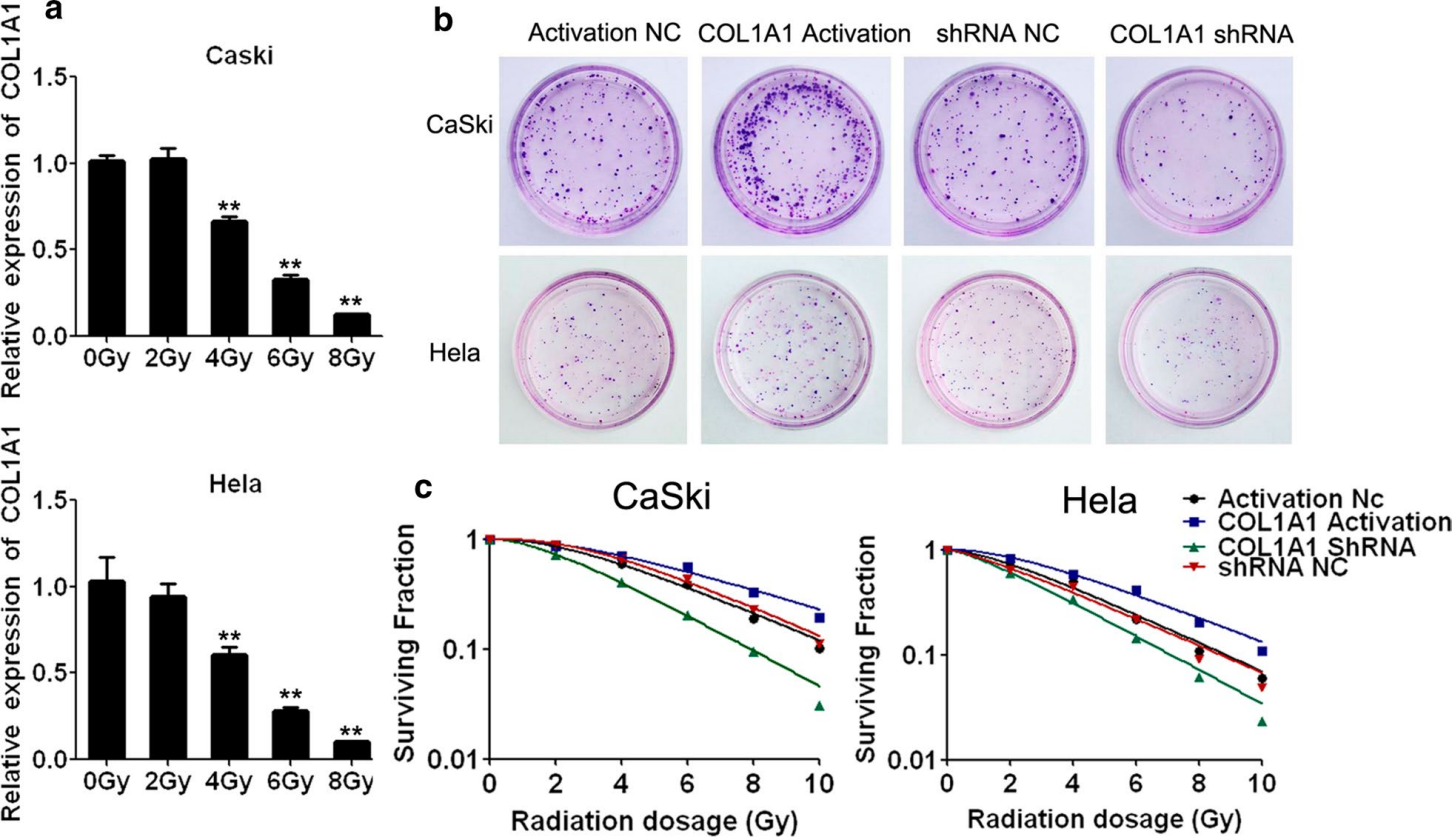
The effects of COL1A1 in cervical cells after treating with radiation. **a** The expression of COL1A1 depended on the the increasing dose of radiation. *P < 0.005 for 4 Gy group vs 0 Gy group in Hela and CaSki cells. **b** Colony formation of different cells to X-ray radiation was detected. The images of the colonies were stained with 0.5% crystal violet in CaSki and Hela cells. **c** Survival fraction of different cells to a dose of X-ray radiation (0–10 Gy). **P < 0.01 vs 0 Gy

As indicated in Fig. 4a, the mRNA expression level of COL1A1 showed a dose dependent decreasing trend with the increasing of radiation in HeLa and CaSki cells. In particular, COL1A1 expression became significantly lower when irradiated with 4 Gy. Thus, 4 Gy was selected for further study.

To further validate the role of COL1A1 in the radioresistance of cervical cancer, COL1A1-shRNA and activation plasmids were used to constructed stably transfected in Hela and CaSki cells, and these cells were irradiated with 4 Gy, then these cells were tested for colony formation (Fig. 4b). In addition, to further validate COL1A1 is related to radioresistance, Hela and CaSki with COL1A1 Activation and COL1A1 shRNA were subjected to X-ray radiation (0, 2, 4, 6, 8 and 10 Gy) (Fig. 4c). As shown in Fig. 4b, after 2 weeks the number of colonies formation in COL1A1-shRNA group was significantly decreased compared with that of negative control (NC) group. However, compared with NC cells, the number of colonies formation were observed significantly increased in Hela and CaSki cells with COL1A1 activation. These results suggested that COL1A1 shRNA cells were more sensitive to radiation than NC cells and COL1A1 Activation cells were more tolerance to radiation than NC cells. Thus, these experiments indicated there is a close relationship between radioresistance and COL1A1 in cervical cells.

### The regulated effect of COL1A1 on apoptosis induced by radiation

**Fig. 5:**
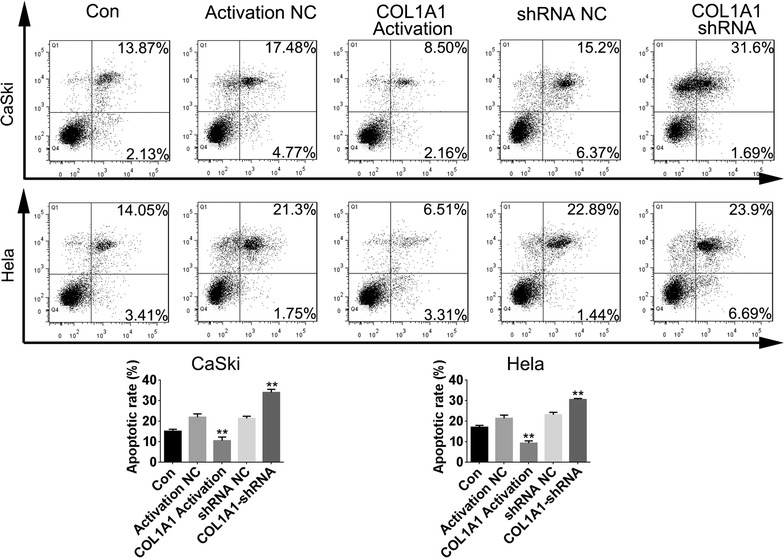
The regulated effect of COL1A1 on apoptosis induced by radiation. In CaSki and HeLa cells, combination of COL1A1 shRNA transfection and radiations induced much more apoptosis than shRNA NC transfection or control cells. Whereas, combination of COL1A1 Activation and radiations group inhibiting apoptosis compared to Activation NC or control group. **P < 0.01 vs control

As shown in Fig. 5, compared with control cell lines, both COL1A1-shRNA and 4 Gy radiation induced significantly apoptosis in Hela and CaSki cells, meanwhile, COL1A1 activation suppressed apoptosis significantly in Hela and CaSki cells compared with NC cells. These data suggested that COL1A1 is important for anti-apoptosis induced by radiation in cervical cancer cells.

### Signaling transduction pathways involved in COL1A1 of anti-apoptosis induced by radiation and the role of COL1A1 in signaling transduction pathways

**Fig. 6:**
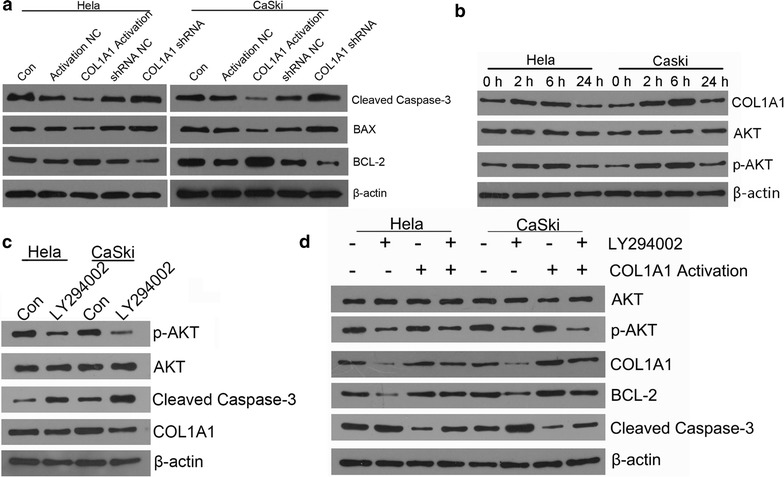
Levels of apoptosis-related proteins are evaluated in cervical cancer cells. **a** Western blotting analysis of Caspase-3, Bax and Bcl-2 expression. The values indicate the COL1A1 may inhibit apoptosis of cervical cancer cells. **b**–**d** Western blotting analysis of individual signal events. The *values* indicate the COL1A1 may be involved in AKT pathway of apoptosis induced by radiation

Since several studies have shown cell apoptosis is associated with the activation of Caspase-3/PI3K/AKT pathway, in our study, western blotting was used to elucidate the mechanism involved in COL1A1 of anti-apoptosis induced by radiation. As shown in Fig. 6a, the COL1A1 activation led to the decreasing of Caspase-3 and Bax, and the increasing of Bcl-2, both in Hela and CaSki cells. However, an opposite tendency was observed in Hela and CaSki cells with COL1A1-shRNA. These results consistently indicated that COL1A1 may inhibit apoptosis of cervical cancer cells.

To further determine the role of COL1A1 in signaling transduction pathways, the expression of apoptosis-related molecules and COL1A1 were measured in Hela and CaSki cells with radiation treated by western blotting (Fig. 6b). As shown in Fig. 6b, X-ray radiation activated the expression of COL1A1, AKT and p-AKT in short time, but suppressed after treated 24 h. At the same time, we used specific inhibitor (LY294002) specifically targeting the pathway to elucidate the mechanism underlying the COL1A1 antiapoptosis activity. As shown in Fig. 6c, LY294002 significantly suppressed the PI3K pathway and led to cell apoptosis, and suppressed the expression of COL1A1. While COL1A1 activation could reverse the cell apoptosis caused by LY294002, and COL1A1 shRNA could enhance the cell apoptosis as shown in Additional file 1: Figure S1 flow cytometry results. To further verify the important role of COL1A1 in signaling transduction pathways of apoptosis in cervical cancer cells with radiation. LY29400 and X-ray radiation were used to treat cervical cancer cells with COL1A1 activation. It was found that COL1A1 reversed the inhibiting effect of LY294002 on apoptosis induced by X-ray radiation (Fig. 6d).

## Discussion

Recent studies have indicated overexpression of COL1A1 as an important event in a series of cancers. Aberrant COL1A1 expression has been found in some solid tumors including gastric and breast [11, 12, 15]. Furthermore, COL1A1 expression may represent an independent biomarker for the prediction of prognosis of hepatocellular carcinoma [15].

Although the important roles of COL1A1 in tumor progression in multiple cancers are now being clarified, it has not been studied in development of cervical cancer. In the present study, we evaluated the possibility of COL1A1 as a therapeutic target of cervical cancer. First evidence from our group demonstrated, the expression of COL1A1 up regulation at mRNA and protein levels from grade I to III in cervical cancer tissues, in contrast to normal cervical tissues. Similarly, previous study also found that the expression of COL1A1 is increased in ovarian serous carcinoma compared normal tissues [16].

It is well established that radiation sensitivity mechanisms in human cancers are determined by intracellular factors and interactions of cells with ECM or neighboring cells as well as the tumor microenvironment [3, 17, 18]. Meanwhile, COL1A1 is a member of collagen family and mainly expressed in the ECM [7–10], and collagens are overexpressed in the majority of human cancers. Thus, these finding indicated that COL1A1 is a radioresistance gene and play an important role in cellular defense irradiation [13]. But the mechanisms that how COL1A1 regulate radioresistance remain elusive. To address this question, we detected the levels of COL1A1 in Hela, CaSki and Siha cells. Interestingly, significant difference was observed among three cell lines, especially for Siha cells. This is likely because of the different radiosensitivity in cells. Furthermore, COL1A1-shRNA and activation cell models were constructed, and we demonstrated that abundant COL1A1 could inhibit the cell death while low-abundant COL1A1 could promote cell death. The findings are further supported by apoptosis analysis. Our findings are consistent with results from other researchers, who also showed that cervical cells enhance radiosensitivity with lower colony survival and higher apoptosis in response to radiation [4, 5, 19].

Apoptosis as an ordered cellular process is of vital importance in regulating cell death [20] so that activation of apoptosis is widely considered as an anticancer strategy. Regarding to the molecular mechanism which leading to the COL1A1 overexpression in cervical cancer, we are thinking that COL1A1 might be induced by activation of radiation dependent Caspase-3/PI3K/AKT signaling pathway. In our study, the expression of several critical apoptosis-related proteins, such as the Bcl-2 family proteins, AKT and Caspase-3, and COL1A1 were detected. Apoptosis regulator Bcl-2 is a family of regulator proteins that regulate cell death (apoptosis), by either inducing (pro-apoptotic) or inhibiting (anti-apoptotic) apoptosis. Bcl-2 is specifically considered an important anti-apoptotic protein [21, 22] and Bax has a pro-apoptotic effect [23]. Caspase-3 plays a central role in the execution-phase of cell apoptosis and is considered as the most important performer of apoptosis in the caspase family [24]. Caspase-3 is activated in the apoptotic cell both by extrinsic (death ligand) and intrinsic (mitochondrial) pathways and caspase activity would kill cells indiscriminately [25, 26]. Subsequently, a significantly decreased expression of p-AKT and Bcl-2 increased expression of Caspase-3 were shown in the LY294002 plus radiation group, compared with radiation alone. Meanwhile, a significantly increased expression of p-AKT and Bcl-2 and decreased expression of Caspase-3 were shown in the COL1A1 activation plus radiation group, compared with radiation alone. However, there was no significant difference in the p-AKT, Bcl-2 and Caspase-3 expression level between the COL1A1 Activation plus LY294002 plus radiation group and radiation alone group. Therefore, we can get the conclusion that COL1A1 Activation may decrease apoptosis of cervical cancer cells by destroying the balance between anti-apoptotic and pro-apoptotic proteins and activating Caspase-3/PI3K/AKT pathway.

What’s more, COL1A1 is a fibril-forming collagen found in most connective tissues and is abundant in bone, cornea, dermis and tendon, and its expression level is closely related to epithelial-to-mesenchymal transition (EMT) [27, 28]. While the EMT was known to be associated with radioresistance [29–31] and PI3K pathway [32–35]. Thus, these indicated that the radioresistance function of COL1A1 may be closely related to EMT.

As a conclusion, COL1A1 seems to modulate the radioresistance of cervical cells via complex mechanisms by affecting Caspase-3/PI3K/AKT pathways to regulate cell death. Afterwards we will try to detect the COL1A1-related radioresistance in animals and the protein that interacted with COL1A1 and the relationship among COL1A1, EMT and radioresistance.

## Conclusions

This is the only study to profile that COL1A1 is a crucial radioresistance factor in cervical cancer cells and plays an important role in ant-apoptosis by Caspase-3/PI3K/AKT pathways. Therefore, our identification of radioresistance-related COL1A1 in cervical cancer should be a starting point to explore the function of collagens, adding a new dimension to our understanding of the complex picture of cervical cancer and assisting cancer biologists and clinical oncologists in designing and testing novel therapeutic strategies.

## Additional file

**Additional file 1: Figure S1.** The regulated effect of COL1A1 on apoptosis tested by flow cytometry. In CaSki and HeLa cells, both COL1A1 shRNA transfection and LY294002 could induce apoptosis. COL1A1 activation could inhibit the apoptosis caused by LY294002, and the combination of COL1A1 shRNA transfection and LY294002 could induce much more apoptosis.

## Abbreviations

COL1A1: collagen type I alpha 1
ECM: extracellular matrix
EMT: epithelial-to-mesenchymal transition
IHC: immunohistochemistry
NC: negative control
qRT-PCR: quantitative real time-polymerase chain reaction
RT: radiotherapy
TMA: tissue microarray
