# Supplementary figures and images for: Regulatory effects of COL1A1 on apoptosis induced by radiation in cervical cancer cells
Source: Cancer Cell Int. 2017 Jul 28;17:73. doi: 10.1186/s12935-017-0443-5 (PMC5534093; doi:10.1186/s12935-017-0443-5)

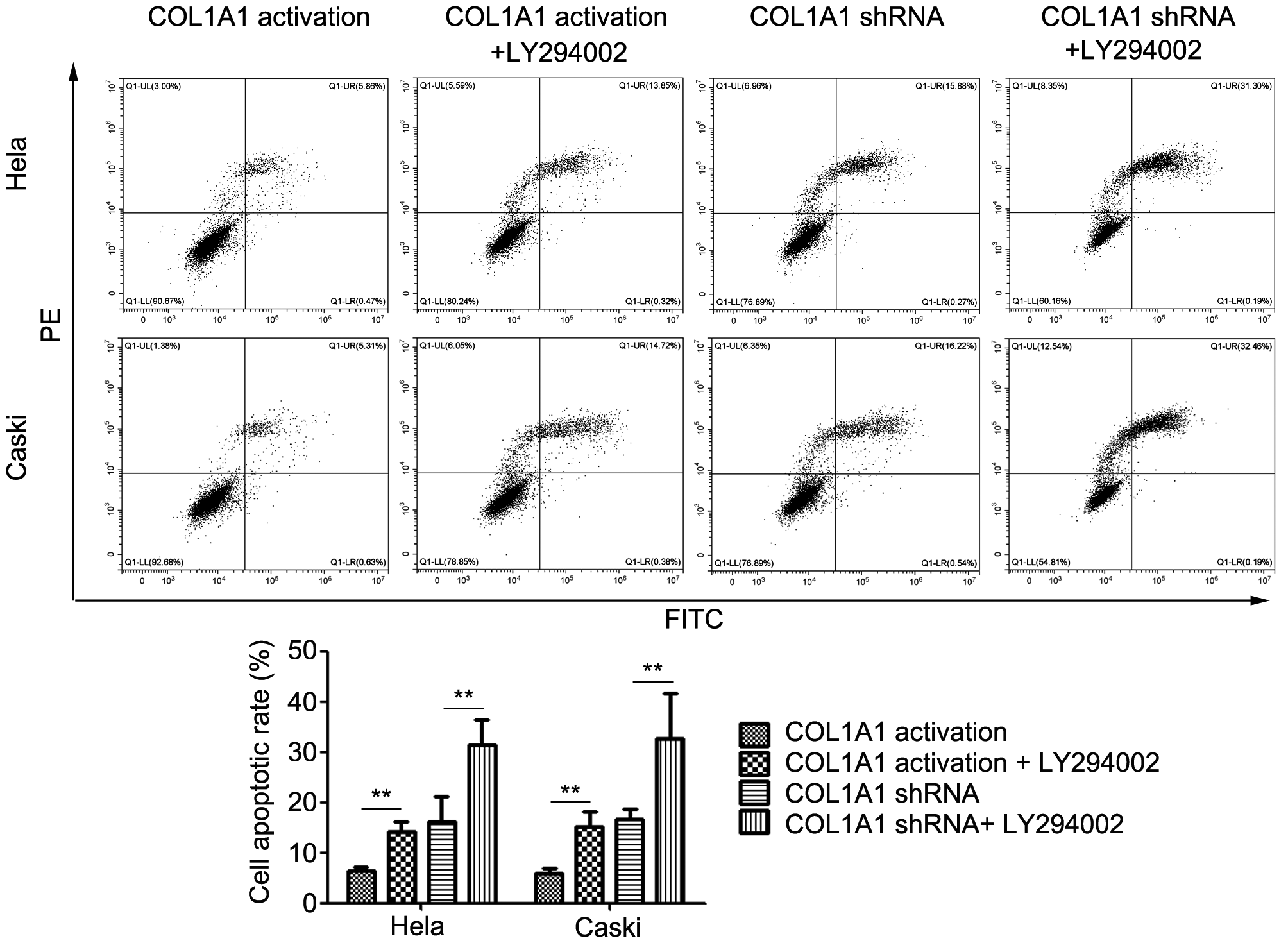

Supplement: Supplementary file 1 — Additional file 1: Figure S1. The regulated effect of COL1A1 on apoptosis tested by flow cytometry. In CaSki and HeLa cells, both COL1A1 shRNA transfection and LY294002 could induce apoptosis. COL1A1 activation could inhibit the apoptosis caused by LY294002, and the combination of COL1A1 shRNA transfection and LY294002 could induce much more apoptosis. [file 12935_2017_443_MOESM1_ESM.tif]
